# The potential role of migratory birds in the transmission of pathogenic *Campylobacter* species to broiler chickens in broiler poultry farms and live bird markets

**DOI:** 10.1186/s12866-023-02794-0

**Published:** 2023-03-10

**Authors:** Maram M. Tawakol, Nehal M. Nabil, Abdelhafez Samir, Hawash H. M., Ahlam E. Yonis, Momtaz A. Shahein, Mona M. Elsayed

**Affiliations:** 1 Reference Laboratory for Veterinary Quality Control On Poultry Production, Animal Health Research Institute, Agricultural Research Center (ARC), Nadi El-Seid Street, Dokki, Giza 12618 Egypt; 2 Animal Health Research Institute, Agricultural Research Center (ARC), Nadi El-Seid Street, Dokki, Giza 12618 Egypt; 3 Department of Hygiene and Zoonoses, Faculty of Veterinary Medicine, Mansoura University, Mansoura, 35516 Egypt

**Keywords:** Campylobacter spp, Broiler chicken, Migratory birds, Poultry farms

## Abstract

**Background:**

*Campylobacter* species (spp.) are one of the most important zoonotic bacteria possessing potential hazards for animal and human health worldwide. Migratory birds are implicated as significant carriers for microbes and a play very important role in the dissemination of *Campylobacter* to broiler chickens and their environment. The purpose of this investigation was to detect the prevalence, antibiotic resistant patterns, virulence and diversity of pathogenic *Campylobacter spp.* in 7 migratory bird species (Northern shoveler, Common pochard, Common teal, Northern pintail, Eared Grebe, Great Crested Grebe and Garganey) and broiler chickens that were collected from broiler poultry farms and live bird markets.

**Results:**

The prevalence of *Campylobacter was* 12.5% (25/200), of which 15% (15/100) was recovered from 5 migratory bird species only and 10% (10/100) from broiler chickens. At the level of migratory birds, eight isolates (53.3%) were *Campylobacter jejuni* (*C. jejuni*) and 7 isolates (46.7%) were *Campylobacter coli* (*C. coli*) meanwhile, in broiler chickens *C. jejuni and C. coli* were 50% (5/10) for each. All isolated strains had phenotypic resistance to doxycycline, while all of the isolates were susceptible to amikacin. The multidrug resistance to three, four or five antimicrobial classes was found in 72% (18/25) of the isolated strains. The multiantibiotic resistance index between the examined isolates was 0.22–0.77, with 10 antibiotic resistance patterns. The virulence of isolated *Campylobacter* strains (from both migratory birds and broiler chicken birds) was detected by targeting the *Vir*B11, *cia*B and *iam* genes which were recorded at 16%, 52% and 100%, respectively. Additionally, 100% and 84% of the antibiotic resistance genes were identified as tetA and BlaOXA-61, respectively.

**Conclusions:**

The results of this study revealed the diversity between all the isolated strains from migratory birds and their similarity to broiler chicken isolates. The findings of the present study highlight the impact of migratory birds visiting Egypt and other countries on pathogenic *Campylobacter spp.* carrying pathogenic virulence and resistance genes, necessitating the application of biosecurity measures to prevent migratory birds from entering farms during their migration period.

**Supplementary Information:**

The online version contains supplementary material available at 10.1186/s12866-023-02794-0.

## Background

*Campylobacter infections* are one of the most common causes of food poisoning, so they have public health and economic importance worldwide [1, 2]. It produces food-borne diarrhea, especially in children and people of old age [3], and approximately 550 million cases suffer from *Campylobacter* infections globally, with lethality of approximately 33 million per year [4]. The mortality rate of children suffering from diarrhea is still above 25% in many African and Southeast Asian countries [5].

Currently, there are approximately 53 species and 16 subspecies of *Campylobacter* [6], among these species, thermophilic *Campylobacter* such as *Campylobacter. jejuni* (*C*. *jejuni)* and *Campylobacter coli* (*C. coli*) which are considered pathogenic to humans and livestock [4].

The main and known sources of human campylobacteriosis are broiler chickens, cattle and their products in addition to the contaminated drinking water [7, 8]. The most of human campylobacteriosis cases are caused by the direct contact with live animals, the handling of contaminated broiler chickens’ meat [9] or indirect contact through the consumption of contaminated poultry or bovine meat [10] and untreated drinking water [11]. However, other risk factors exist, such as wild birds and the epidemiology of this disease in wild birds and its transmission from wild birds to domestic birds and animals have not yet been clearly detected. Currently, the available information about the role of wild birds as sources and reservoirs of *Campylobacter* is limited, which may be related to the difficulty in the collection of samples from wild birds [12].

*Campylobacter* is found in the intestinal tracts of avian species including poultry [13] and wild birds [14] as common commensals and is distributed widely in the aquatic environments whereas *Campylobacter* spp. have the ability to survive and remain for a long time as potentially pathogenic [15]. Other study recorded that *C. jejuni* can induce chronic inflammation, damage to gut tissue, and diarrhoea in broiler chicks in addition to being a commensal bacterium [16]. *Campylobacter* colonization in farm chickens typically results by horizontal environmental transfer, such as through feed or drinking water. Just *Campylobacter* enters the flock of chickens, it spreads quickly and, after one week, has colonized the intestinal tracts (ceca, small intestine, and crap) of the majority of birds [17–19]. Additionally, the transportation of live birds from farms to processing facilities has been noted as a crucial harbor for the spread and colonisation of *Campylobacter* [19]. Therefore, migratory birds are carriers of most pathogenic microbes, either biologically or mechanically [20], and disseminate antimicrobial resistant pathogens (to domestic animals and birds or various environmental water sources) during their migration period through fecal droppings [14]. They are a well-known significant natural reservoir of *C*. *jejuni, C. coli, and C. lari* [12].

*Campylobacter* strains have been observed to become more resistant over time to the drugs of choice including fluoroquinolones and macrolides and alternative therapies including gentamicin and tetracycline [21], making *Campylobacter* strains that are antibiotic resistant a public health threat. [22]. The abuse of antibiotics in both human and animal agriculture is a contributing factor in the development of antimicrobial resistance. For instance, the widespread use of various quinolone antibiotics in poultry farming in Africa, China, Europe and Italy during the period from 2008 until 2015 has resulted in an increase in the number of *Campylobacter* strains that are resistant to quinolones in both chickens and humans [23]. Compared to other enteric bacteria, *Campylobacter* species have a virulome that is used in attachment, establishment, invasion, and toxin production, leading to their high prevalence [24, 25], which represents a potential hazard for both animal and human health [26]. One of the major pathogenicity factors of *C. jejuni* is the cytolethal distending toxin (*cdt* A, B, C) complex, which helps induce host cell apoptosis. The flagellum of this bacterium plays the main role in motility and secretion of invasive antigens [27].

Migratory birds cross national and international borders [28]. Egypt is considered a transit point for bird flocks migrating from Europe to Africa. Millions of birds travel across Egypt in search of food, rest and shelter [29, 30]. These migratory flocks are drawn to Egypt by its moderate winter weather and proximity to the Red Sea, which ensure ample food supplies. Wild birds have been observed in abundance in residential areas, farms and marketplaces, so these birds can transmit *Campylobacter spp.* to animal [31] and poultry farms [3] through their droppings and/or picking up bacteria from farm litter and contaminated poultry houses [32]. This work for the first time investigated the role of migratory birds as disseminators for pathogenic *Campylobacter spp.* to broiler chicken birds in broiler poultry farms and live bird markets in Egypt. In this study, we determined the prevalence, antibiotic resistance patterns, virulence genes and diversity of pathogenic *Campylobacter spp.* (*C. jejuni* and *C. coli*) in migratory and broiler chickens.

## Results

### The prevalence of *campylobacter* spp. in the examined migratory and broiler chickens

The prevalence and species of *Campylobacter* were screened in this study in 100 cloacal swabs from migratory birds and 100 from broiler chicken birds. At the level of migratory birds, 100 cloacal swabs were collected from that were found near the examined farms and live bird markets at which 7 different species were detected, including Northern shoveler (Spatula clypeata) (*n* = 21), Common pochard (Aythyaferina) (*n* = 22), Common teal (Anas crecca) (*n* = 20), Northern pintail (Anas Acuta) (*n* = 16), Eared Grebe (Podiceps nigricolls) (*n* = 9), Great Crested Grebe (Podiceps cristatus) (*n* = 3) and Garganey (Spatula querquedula) (*n* = 9). Out of 100 samples, *Campylobacter* spp. were detected in 15 (15%) samples, of which 8 isolates (53.3%) were *C. jejuni* and 7 (46.7%) were *C. coli* (Table 1) (Supplementary Fig. 1), including 5 species of the examined migratory birds (Common teal, Northern pintail, Common pochard, Northern shoveler and Great crested grebe). At the level of birds spp., *Campylobacter* strains were observed in 9.5% (2/21) of Northern shoveler birds (*C. jejuni* and *C. coli;* one for each), 13.6% (3/22) of Common pochard birds (*C. jejuni*), 25% (5/20) of Common teal birds (2; *C. jejuni* and 3; *C. coli*), 25% (4/16) of Northern pintail birds (1; *C. jejuni* and 3; *C. coli*), 33.3% (1/3) of Great crested grebe birds (*C. jejuni*). Meanwhile, Eared grebe and Garganey birds were negative for *Campylobacter* spp. No mixed infections with *C. jejuni* and *C. coli* were detected in this study.* C. jejuni* was detected in the five migratory bird species, while *C. coli* was found only in 3 species only (Common teal, Northern pintail, and Northern shoveler).

**Table 1:** Prevalence of *Campylobacter* species among the examined migratory and domestic birds

**Birds type**	**Bird species** **(Scientific name)**	**Total No. of samples**	**No. of Positive samples (%)**	***Campylobacter* spp.**	
				No. of* Campylobacter jejuni*	No. of* Campylobacter coli*
**Migratory birds**	Northern shoveler (*Spatula clypeata)*	21	2 (9.5)	1 (50)	1 (50)
	Common pochard (*Aythya ferina*)	22	3 (13.6)	3 (100)	0
	Common teal (*Anas crecca)*	20	5 (25)	2 (40)	3 (60)
	Northern pintail (*Anas Acuta*)	16	4 (25)	1 (25)	3 (75)
	Eared Grebe (*Podiceps nigricollis*)	9	0	0	0
	Great Crested Grebe (*Podiceps cristatus*)	3	1 (33.3)	1 (100)	0
	Garganey (*Spatula querquedula*)	9	0	0	0
**Total**		**100**	**15 (15)**	**8 (53.3)**	**7 (46.7)**
**Broiler chicken**	Broiler from poultry farms	50	6 (12)	3 (50)	3 (50)
	Broiler from live bird markets	50	4 (8)	2 (50)	2 (50)
**Total**		**100**	**10 (10)**	**5 (50)**	**5 (50)**

In broiler chickens, cloacal swabs were collected randomly from birds in broiler chicken farms and live bird markets (50 for each) and the migratory birds were found near it. The prevalence of *Campylobacter* was 12% (6/50) in birds from poultry farms and 8% (4/50) in birds from live bird markets. In birds from poultry farms, *C. jejuni* and *C. coli* were 50% (3/6) for each and birds from live bird markets were 50% (2/4) for each.

### Antimicrobial resistance testing

The antimicrobial resistance of the recovered *Campylobacter* strains against 9 types of antibiotics, which included 5 different classes revealed absolute resistance to doxycycline (DO) (100%) followed by tetracycline (TE), erythromycin (E) (80% for each), ciprofloxacin (CIP) (72%), streptomycin (S) (52%), amoxicillin (AX) and ampicillin (AM) (48% for each). Meanwhile, norfloxacin (NOR) showed a lower rate of resistance (8%). It is interesting to note that none of the isolates have AK resistance (Table 2). Additionally, there was a difference between *C. jejuni* and *C*. *coli* about antibiotic resistance and MDR. None of *C. jejuni* isolates were resistant to NOR while, two isolates of *C*. *coli* were resistant to it. *C. jejuni* showed higher resistance rates to TE, E, CIP, S and AM (84.6%, 92%, 76.9%, 53.8% and 61.5%, respectively) than *C. coli* (75%, 66.7%, 66.7%, 50% and 25%, respectively) (Table 2). In addition, 72% (18/25) of isolates had multidrug resistance (MDR) to three, four or five antimicrobial classes, with a multiple antibiotic resistance index (MARI) of 0.55–0.77. *C. jejuni* represented 10 isolates out of 18 that were MDR to four or five different classes of examined antibiotics while, *C. coli* represented 8 isolates that were MDR to only three or four different classes of antibiotics (Table 3). *Campylobacter* strains in this study demonstrated 10 distinct antibiotic resistance patterns (Table 3), reflecting the high prevalence of MDR among *Campylobacter* strains in the examined migratory birds.

**Table 2:** Antibiotics Susceptibility pattern of the isolated *Campylobacter spp.*

**Classes of antibiotics**	**Antibiotics**	***Campylobacter* spp. (25 isolates)**			***Resistant isolates***	
		No. of sensitive isolates (%)	No. of intermediate isolates (%)	No. of resistant isolates (%)	No. of C. *jejuni* (%)	No. of *C. coli* (%)
					13	12
Penicillin	AX	8 (32)	5 (20)	12 (48)	6 (46)	6 (50)
	AM	3 (12)	10 (40)	12 (48)	8 (61.5)	4 (25)
Macrolides	E	2 (8)	3 (12)	20 (80)	12 (92)	8 (66.7)
Aminoglycosides	AK	23 (92)	2 (8)	0	0	0
	S	5 (20)	7 (28)	13 (52)	7 (53.8)	6 (50)
Tetracyclines	DO	0	0	25 (100)	13 (100)	12 (100)
	TE	0	5 (20)	20 (80)	11 (84.6)	9 (75)
Fluoroquinolones	CIP	0	7 (28)	18 (72)	10 (76.9)	8 (66.7)
	NOR	20 (80)	3 (12)	2 (8)	0	2 (12.5)

*AX* Amoxicillin, *AM* Ampicillin, *E* Erythromycin, *S* Streptomycin, *AK* Amikacin, *TE* Tetracyclines, *DO* Doxycycline, *NOR* Norfloxacin, *CIP* Ciprofloxacin

**Table 3:** Antibiotic pattern profiles of isolated *Campylobacter* strains

Antibiotic pattern profiles	Antibiotics	No. of resistant isolates	No. of resistance antibiotics	MARI
1	**DO, TE, E, CIP, S, AM, AX**	**4 (3; *C. jejuni* and 1; *C. coli*)**	**7**	**0.77**
2	**DO, TE, CIP, S, AM, AX, NOR**	**2 (*C. coli*)**	**7**	**0.77**
3	**DO, TE, E, CIP, AM, AX**	**3 (*C. jejuni*)**	**6**	**0.66**
4	**DO, TE, E, CIP, S, AX**	**2 (*C. coli*)**	**6**	**0.66**
5	**DO, TE, E, CIP, S, AM**	**2 (*C. jejuni*)**	**6**	**0.66**
6	**DO, TE, E, CIP, S**	**3 (2; *C. jejuni* and 1; *C. coli*)**	**5**	**0.55**
7	**DO, TE, E, CIP**	**2 (*C. coli*)**	**5**	**0.55**
8	**DO, AX, AM**	**1 (*C. coli*)**	**3**	**0.33**
9	**DO, TE**	**2 (1: *C. jejuni* and 1; *C. coli*)**	**2**	**0.22**
10	**DO, E**	**4 (2: *C. jejuni* and 2; *C. coli*)**	**2**	**0.22**

*AX* Amoxicillin, *AM* Ampicillin, *E* Erythromycin, *S* Streptomycin, *AK* Amikacin, *TE* Tetracyclines, *DO* Doxycycline, *NOR* Norfloxacin, *CIP* Ciprofloxacin

### Distribution of species specific, virulence and antimicrobial resistance genes among the *campylobacter* isolates

In this study, the species of isolated *Campylobacter* was detected by targeting 2 species specific genes (*map*A for *C. jejuni* and *ceu*E for *C. coli*), 3 virulence genes (*Vir*B11, *cia*B and *iam*) and 2 antimicrobial resistance genes (*tet*A and BlaOXA-61). These 3 pathogenic virulence genes are responsible for *Campylobacter* invasion in host cells. Generally, *map*A and *ceu*E genes were identified in 13/25 (52%) and 12/25 (48%) of the isolates, respectively (Table 4) (Supplementary Figs. 2 and 3). The majority of *Campylobacter* isolates (19/25) displayed at least two virulence-associated genes. (Table 4). Of note, the *iam* gene was found in all isolates (100%, 15/15), while the *cia*B and *Vir*B11 genes were detected in 52% (13/25) and 16% (4/25) of the isolates, respectively (Table 4) (Supplementary Figs. 4, 5 and 6). For the antibiotic resistance genes, the *tet*A gene was investigated in all isolates (100%) and *Bla*OXA-61 genes were observed in 84% of the isolates (21/25) (Table 4) (Supplementary Figs. 7 and 8).

**Table 4:** Distribution of Species specific, virulence and antimicrobial resistance genes among the isolated *Campylobacter* spp

Isolate Code	Bird Spp	*Species specific genes*		Antibiotics resistance patterns	*Virulence genes*			*Antibiotics resistant genes*	
		*map*A for *C. jejuni*	*ceuE for C. coli*		***VirB11***	***ciaB***	***iam***	***tetA***	***BlaOXA-61***
1	Northern shoveler	-	+	2	-	-	+	+	+
2	Northern shoveler	+	-	1	-	+	+	+	+
3	Common teal	-	+	1	+	-	+	+	-
4	Common teal	-	+	9	+	-	+	+	+
5	Common teal	-	+	6	-	-	+	+	+
6	Northern pintail	-	+	4	-	+	+	+	+
7	Northern pintail	-	+	10	-	+	+	+	+
8	Pochard	+	-	10	-	-	+	+	-
9	Northern pintail	+	-	10	-	-	+	+	+
10	Common teal	+	-	3	+	-	+	+	+
11	Common teal	+	-	1	-	-	+	+	+
12	Common pochard	+	-	3	-	+	+	+	-
13	Common pochard	+	-	5	-	+	+	+	+
14	Great Crested Grebe	+	-	6	-	+	+	+	+
15	Northern pintail	-	+	7	-	-	+	+	+
16	Broiler poultry (farms)	-	+	10	-	+	+	+	+
17	Broiler poultry (farms)	+	-	1	-	-	+	+	+
18	Broiler poultry (farms)	-	+	7	-	-	+	+	+
19	Broiler poultry (farms)	+	-	3	-	+	+	+	-
20	Broiler poultry (farms)	+	-	9	+	-	+	+	+
21	Broiler poultry (farms)	-	+	2	-	+	+	+	+
22	Broiler poultry (markets)	-	+	4	-	+	+	+	+
23	Broiler poultry (markets)	+	-	5	-	+	+	+	+
24	Broiler poultry (markets)	+	-	6	-	+	+	+	+
25	Broiler poultry (markets)	-	+	8	-	+	+	+	+

1; DO, TE, E, CIP, S, AM, AX, 2; DO, TE, CIP, S, AM, AX, NOR, 3; DO, TE, E, CIP, AM, AX, 4; DO, TE, E, CIP, S, AX, 5; DO, TE, E, CIP, S, AM, 6; DO, TE, E, CIP, S, 7; DO, TE, E, CIP, 8; DO, AX, AM, 9; DO, TE, 10; DO, E

**Fig. 1:**
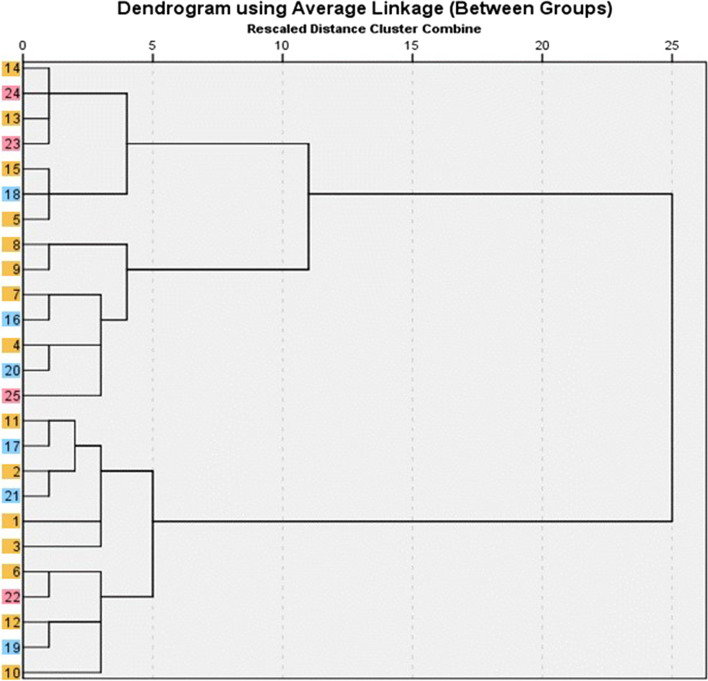
Dendrogram for the detection of similarity and diversity between the isolated strains (1:15 was from migratory birds, 16:21 from boiler poultry farms and 17:25 from Live bird markets). Isolates from migratory birds, broiler poultry farms and live bird markets were highlighted with orange, blue and pink color, respectively.

The results in table (4) (Fig. 1) show that all isolated *Campylobacter* strains from migratory birds were diverse in their profiles (antibiotic resistance patterns, virulence genes and antibiotic resistance genes), even those isolated from the same species of migratory birds. For example, isolates with codes of 1 and 2 were isolated from the same species of migratory birds (Northern shoveler) but differed in the level of species-specific genes, antibiotic resistance pattern and virulence gene profile (Table 4). On the other hand, isolates with codes of 3 and 4 were isolated from the same species of migratory birds (common teal) and were similar in the level of species-specific genes, antibiotic resistance pattern and virulence gene profile but different in the presence of the *BlaOXA-61 gene* (Table 4). Meanwhile, there was similarity (80%; 8/10) between the *Campylobacter* strains from broiler chickens and those from migratory birds (Table 4) (Fig. 1). Examples include isolate No. 4 with isolate No. 20, isolate No. 6 with isolate No. 22, isolate No. 7 with isolate No. 16, isolate No. 11 with isolate No. 17, isolate No. 13 with isolate No. 23, isolate No. 14 with isolate No. 24, isolate No. 15 with isolate No. 18 and isolate No. 12 with isolate No. 19 from migratory birds and broiler chickens, respectively.

## Discussion

*Campylobacter* commonly inhabit the intestinal tract of avian and wild birds [33], so migratory wild birds could play a significant role in the dissemination and transmission of *Campylobacter* spp. [34] to farm animals and their environments, especially poultry farms [31, 32]. In the current study, *Campylobacter* was isolated from 12.5% (25/200) of the collected samples for both migratory and broiler chicken birds. At the level of bird type, samples were collected from 5 migratory bird species (common teal, northern pintail, common pochard, northern shoveler and great crested grebe) with a prevalence of 15% (15/100); 8 isolates (53.3%) were *C. jejuni,* and 7 isolates (46.7%) were *C. coli*. The recovery of thermophilic *Campylobacter* spp. (*C. jejuni* and *C. coli*), which are crucial global zoonotic pathogens that cause gastroenteritis in humans [4], in this study clearance the important role of migratory birds in the transmission of *Campylobacter* infection to domestic animals and their environment and to humans. The prevalence of *Campylobacter spp*. was lower in this study than that detected by Sensale et al. [34], Kwon et al. [33] and Kürekci et al. [35], who isolated *Campylobacter* from Eurasian coots in Turkey (93%), waterfowl in Washington (73%), birds in the Campania region (23.1%) and winter migratory Stopover birds (16.7%). On the other hand, a lower prevalence was detected in the southern Sweden coasts by Waldenström et al., [36] who isolated *C. jejuni* (5.0%), *C. lari* (5.6%) and *C. coli* (0.9%) from migratory birds. The variation in the isolation percentages may be attributed to some factors that have a role in *Campylobacter* transmission, such as wild bird species, geographical location, sampling time, migration patterns (foraging on the ground close to animal farms, foraging far from the farms of animals, or hunting in the air) and stress factors during the migration period [12, 13]. Additionally, the size of the bird has a role in the probability of carrying *Campylobacter* infection, whereas the smaller and larger birds differ in their habitat preference and distributions [36]. Wild birds move over large distances, and many species of birds are concentrated at wintering, breeding and stopover places, which help pathogen transmission between different species [37]. Furthermore, stress factors during migration movement may increase the susceptibility of infections, and pathogen shedding increases, resulting in water and soil contamination with fecal matter and helping the transmission of pathogens to new environments [34].

In broiler chickens, the prevalence of *Campylobacter spp.* was 12% (6/50) in birds from poultry farms and 8% (4/50) in birds from live bird markets. In the birds from poultry farms,* C. jejuni* and *C. coli* were 66.7% (4/6) and 33.3% (2/6), respectively. Birds from live bird markets were negative for *C. coli*, while *C. jejuni* represented 100% (4/4) of the isolates. Franciska et al. [38] found a higher prevalence in cecal samples, which was 97% at layer farms and 93% at broiler farms with *Campylobacter*-positive flocks with *C. jejuni* (40%) and *C. lari* (7%) in layers and *C. jejuni* (100%) in broilers**.** Additionally, the high prevalence of *Campylobacter* spp. was primarily found in cloacal swabs (21/49, 43%) from broiler poultry farms and in broiler meat (14/26, 54%) from live bird markets in Bangladesh [39]. This study showed a low prevalence rate of *Campylobacter* in birds from poultry farms that contributed to the sampling period, which was conducted between October and March (cold months). The lowest prevalence of *Campylobacter* in broiler farms in Sweden was found during the sampling period falls in the cold months (January and May), while the highest prevalence was recorded in the month (August) with the highest temperature during sampling days [40].

One of the growing worldwide health issues is antibiotic resistance [41]. Referring to the recorded results of the antimicrobial susceptibility testing of 25 isolates against 9 antibiotics, higher resistance was recorded to DO (100%), E and TE (80% for each), while higher susceptibility was recorded to AK (92%) and NOR (80%). Our findings differ from previous studies conducted by [33], who recorded lower resistance to TE (1.8%), and [25], who reported resistance to CIP, TE and E (33.3%, 23.3% and 23.3%, respectively). On the other hand [12], displayed resistance of *Campylobacter* to CIP, TE and S at 69.9%, 55.6% and 6.7%, respectively.

Multidrug resistance was noticed in most *Campylobacter* isolates in this study, with MDRI ranging from 0.22 to 0.77. Globally, antimicrobial resistance represents a major challenge in human and animal health. Both wild birds and animals are not directly exposed to antimicrobial agents [42], and they can acquire antimicrobial-resistant bacteria from contaminated habitats and environments during their movements. Additionally, these birds have the ability to spread resistant bacteria again during their migration to humans through direct contact with their droppings and to animal and poultry farms via deficiencies in biosecurity programs.

The results showed the detection of the *map*A gene in 13 (52%) isolates of *C. jejuni* and the ceu*E* gene in 12 (48%) isolates of* C. coli,* which have an important role in virulence and regulate the siderophore transport system. Pathogenic virulence genes responsible for the expression of the invasion of *Campylobacter* in host cells were reported as follows: *Vir*B11 (16%), *cia*B (52%) and *iam* (100%). A study performed by Shyaka et al. [26] isolated *C. jejuni* from wild birds in Japan and recorded virulence genes (*cdt*A, *cdt*B and *cdt*C), cytotoxin genes (*fla*A, *fla*B and *cad*F) and the *cia*B gene (associated with invasion). Additionally, another study was conducted by Wei et al. [43] and reported *cad*F, *fla*A, *cdt*B, *cdt*C, *vir*B11 and *wla*N genes in *Campylobacter* spp. isolated from wild birds in South Korea. In the current study, *the tet*A and *Bla*OXA-61 genes were identified with percentages of 100% and 84%, respectively. These findings agreed with those of Marotta et al. [44], who detected blaOXA-61 in *C. jejuni* isolated from wild birds in Italy. In this study, the distribution of pathogenic virulence genes (*Vir*B11, *cia*B and *iam*) and antibiotic resistance genes (*tet*A and *Bla*OXA-61) represents a public health concern because migratory birds are reservoirs for pathogenic microbes.

The diversity of species-specific genes, antibiotic resistance patterns, virulence genes and antibiotic resistance genes between all 15 isolates of Campylobacter isolated from migratory birds indicates the possibility of migratory birds playing an important role in the transmission of different strains from different localities. On the other hand, the similarity between the isolates of *Campylobacter* from broiler chicken birds (from poultry farms and live bird markets) and those of migratory birds suggested that migratory birds were the main source of *Campylobacter* to domestic birds inside the farms and live bird markets. Additionally, it is an indication of the difficulties of infection control, so it necessitates the importance of migratory bird prevention from the entrance to poultry farms and live bird markets through the application of biosecurity measures [1], as well as wild bird fecal material and secretions from being accidently transported on boots, equipment and food to birds [4]. Through netting, laser bird repellents and foot baths were used at the entrance of the farms.

### Limitations

It should be noted that there are some limitations to the present study. Although this is the first study to investigate the role of migratory birds as disseminators of pathogenic *Campylobacter spp.* to broiler chickens in broiler poultry farms and live bird markets in Egypt, it focused on chicken samples collected during only one season and from only one province of Egypt and did not elucidate their role in other seasons and other provinces. Therefore, additional studies are warranted to explore such profiles in other seasons and other provinces of Egypt and other species of birds. Moreover, future in-depth studies are necessary.

## Conclusions

This study for the first time investigated the role of migratory birds as disseminators for *Campylobacter spp.* to broiler chicken birds in Egypt. *C. jejuni* and *C. coli* strains, which carry virulence and antibiotic resistance genes with diversity between all isolates, were detected in the examined migratory birds (trapped near poultry farms and live bird markets), indicating the high risk of migratory birds in the transmission of different strains from different localities and the difficulties in its control. Additionally, the similarity between the isolates of *Campylobacter spp.* from broiler chicken birds (from poultry farms and live bird markets) and that of migratory birds indicate that migratory birds were the main source of *Campylobacter* to broiler chicken birds inside the farms and live bird markets.

These findings elucidated the importance of biosecurity programs and strict measures to prevent migratory birds from entering farms and live bird markets. Egypt is a transit point for many migratory birds between different continents and habitats, so continuous surveillance programs for migratory birds visiting Egypt should be implemented to collect more details about the epidemiology of these birds in the circulation of *Campylobacter* infections and other zoonotic pathogens between different countries.

## Methods

### Collection and preparation of samples

This study was conducted in Damietta Governorate on the Egyptian Mediterranean coast (northern east Nile Delta), Egypt through the period from October 2021 to March 2022. A total of 200 cloacal swabs were collected from migratory and broiler chicken birds. Broiler chickens were selected from poultry farms and live bird markets near which the migratory birds were hunted at the similar time points. One hundred samples were obtained from migratory birds and 100 from broiler chickens; 50 from 5 poultry farms (10 for each farm) with deep litter system and 50 from 3 live bird markets located in different regions inside Damietta Governorate. Five broiler poultry farms were chosen on the basis of their owners’ willingness to permit the samples collection. Broiler chicken birds from the farms and live bird markets were selected randomly. The map of Damietta Governorate was constructed to highlight the location of the selected broiler chicken farms and live bird markets in relation to the rest of Damietta (Supplementary Fig. 9). The migratory birds that were found near to the examined farms and live bird markets were trapped by net traps, sampled, marked (to ensure that each bird was only sampled once) and photographed to detect its species. The cotton swabs were aseptically collected on 2 ml of Bolton broth (Oxoid, UK) then labeled and transported within 1 h in an ice box at 4 °C to the Reference Laboratory for Veterinary Quality control on Poultry production to perform further examinations. All samples were incubated at 42 °C for 48 h under microaerophilic conditions. Isolation and identification of *Campylobacter* spp.

Each enriched sample was streaked onto modified charcoal cefoperazone deoxycholate agar (Oxoid, UK) with antibiotic solution (cefoperazone sodium salt; 0.032 g, amphotericin B; 0.01 g and water; 5 ml) and incubated at 42 °C for 48 h. The suspected colonies were identified by morphological characteristics and Gram staining [45]. The suspected isolates were subjected to standard biochemical procedures, including tests for hippurate, acetate hydrolysis and catalase [46].

### Molecular characterization of *C. jejuni* and *C. coli*

All biochemically verified isolates were subjected to PCR analysis for the detection of the 23S rRNA gene, which revealed the presence of thermotolerant *Campylobacter* spp., and then for the identification of the species, two differentiation genes (*mapA* for *C. jejuni* and *ceuE* gene for *C. coli*) were used. The QIAamp DNA Mini kit (Qiagen, Germany, GmbH) was used to extract DNA in accordance with the manufacturer's instructions. Briefly, 200 µl of the sample suspension was treated at 56 °C for 10 min with 10 µl of proteinase K and 200 µl of lysis solution. Then, 200 µl of 100% ethanol was added to the lysate after incubation. After that, the sample was washed and centrifuged in accordance with the manufacturer's instructions with the help of 100 µl of elution buffer, and DNA was extracted.

The oligonucleotide primers used in this study were provided by Metabion (Germany) (supplementary table 1). A 25-µl reaction containing 12.5 µl of Emerald-Amp Max PCR Master Mix (Takara, Japan), 1 µl of each primer at a 20 pmol concentration, 5.5 µl of water, and 5 µl of DNA template was used. Thermal cycler 2720 from Applied Biosystems was used to perform the reaction.

The PCR products were separated using 5 V/cm gradient electrophoresis on a 1.5% agarose gel (Applichem, Germany, GmbH) in 1 × TBE buffer at room temperature. Each gel slot had 20 µl of the product for gel analysis. The fragment sizes were calculated using the Generuler 100 bp ladder (Fermentas, Germany) and the Gelpilot 100 bp ladder (Qiagen, Gmbh, Germany). A gel documentation system (Alpha Innotech, Biometra) took pictures of the gel, and computer software was used to analyze the data.

### Antimicrobial susceptibility testing

The in vitro susceptibility of all confirmed *Campylobacter* strains was determined by using the disc diffusion method on Mueller–Hinton agar (Oxoid, UK) according to the guidelines of the Clinical and Laboratory Standards Institute (CLSI) [47]. Antimicrobial agent selection was based on the importance for both human and veterinary fields in addition to their antimicrobial mechanisms. Nine antibiotics belonging to five classes were selected. They included penicillin (AX; 20 μg and AM; 10 μg), macrolides (E; 15 μg), aminoglycosides (S; 10 μg and AK; 30 μg), tetracyclines (TE; 30 μg and DO; 30 μg), fluoroquinolones (NOR; 10 µg and CIP; 5 μg). All antimicrobial agents used in this study were purchased from Oxoid (England). *C. jejuni* ATCC 33,560 and *C. coli* ATCC 33,559 were used as control strains. MDR strains of *Campylobacter* are those that are resistant to three or more different classes of antimicrobials. Additionally, MARI for all *Campylobacter* isolates was calculated using the formula a/b (where "a" represents the number of antimicrobials to which an isolate was resistant and "b" represents the overall number of antimicrobials to which the isolate was exposed) [48].

### Molecular detection of virulence and antibiotic resistance genes

All *Campylobacter* strains were examined using the uniplex PCR technique for *Vir*B11, *cia*B and *iam* virulence genes, which facilitate the invasion of *Campylobacter* inside host cells. Additionally, *tet*A and BlaOXA-61 antibiotic resistance genes for tetracyclins and extended-spectrum β-lactamases, respectively, were detected in all *Campylobacter* strains. In Supplementary Table 1, the primer sequence, cycle conditions, and predicted amplicon size are shown. Both PCR and electrophoresis were carried out as previously mentioned. Saline served as the negative control, and *C. jejuni* ATCC 33,560 and *C. coli* ATCC 33,559 served as the positive controls. 

### Statistical analysis

Version 15.0 of Microsoft Excel was used to record the data, and version 22 of SPSS [Statistical Package for Social Science] was used to conduct the analysis. In the calculation of the prevalence, descriptive statistics such as percentages and frequency distributions were used. The patterns of antibiotic sensitivity were displayed as percentages. Additionally, the dendrogram for cluster analysis was presented using SPSS, version 22.

## Supplementary Information

**Additional file 1: Table 1. **oligonucleotide primers used for *Campylobacter* isolates characterization. **Figure 1. **Representative agarose gel electrophoresis of PCR products for *Campylobacter* isolates to detect *23S rRNA* in genomic DNA. Lane L: DNA ladder, P: Positive control, N: Negative control and Lanes: 1 to 15 were positive samples. **Figure 2.** Representative agarose gel electrophoresis of PCR products for *Campylobacter jejuni* to detect *map*A in genomic DNA. Lane L: DNA ladder, P: Positive control, N: Negative control and Lanes: 2, 8, 9, 10, 11, 12 ,13 and 14 were positive samples and Lanes: 1, 3, 4, 5, 6, 7 and 15 were negative. **Figure 3.** Representative agarose gel electrophoresis of PCR products for *ceu*E detection in genomic DNA. Lane L: DNA ladder, P: Positive control, N: Negative control and Lanes: 2, 8, 9, 10, 11, 12, 13 and 14 were negative samples and Lanes: 1, 3, 4, 5, 6, 7 and 15 were positive. **Figure 4.** Representative agarose gel electrophoresis of PCR products *VirB11* detection in genomic DNA. Lane L: DNA ladder, P: Positive control, N: Negative control and Lanes: 1, 2, 5, 6, 7, 8, 9, 11, 12, 13, 14 and 15 were negative samples and Lanes: 3, 4, and 10 were positive. **Figure 5.** Representative agarose gel electrophoresis of PCR products for *ciaB* detection in genomic DNA. Lane L: DNA ladder, P: Positive control, N: Negative control and Lanes: 1, 2, 3, 4, 5, 8, 9, 10, 11 and 15 werenegative samples and Lanes: 6, 7, 12, 13 and 14 were positive. **Figure 6.** Representative agarose gel electrophoresis of PCR products for *iam *detection in genomic DNA. Lane L: DNA ladder, P: Positive control, N: Negative control and Lanes: 1 to 15 were positive. **Figure 7.** Representative agarose gel electrophoresis of PCR products to detect *tet*Ain genomic DNA. Lane L: DNA ladder, P: Positive control, N: Negative control and Lanes: 1 to 15 were positive. **Figure 8.** Representative agarose gel electrophoresis of PCR products to detect *BlaOXA-61 *in genomic DNA. Lane L: DNA ladder, P: Positive control, N: Negative control and Lanes: 1, 2, 4, 5, 6, 7, 8, 9, 10, 11, 13 and 14 were positive and Lanes: 3, 12 and 15 were negative. **Figure 9. **Map of Damietta Governorate showing the location of the selected five broiler chicken farms (blue circles represents poultry farms) and the three live bird markets (orange circles represents poultry farms) for the study in relation to the rest of Damietta Governorate.

## Data Availability

All data generated or analyzed during this study are included in this published article and its supplementary information files.

## Abbreviations

Ak: Amikacin
AM: Ampicillin
AX: Amoxicillin
*C. jejuni*: *Campylobacter* *jejuni*
*C. coli*: *Campylobacter coli*
CIP: Ciprofloxacin
CLSI: Clinical and Laboratory Standards Institute
DO: Doxycycline
E: Erythromycin
MDR: Multidrug resistant
MARI: Multiple antibiotic resistance index
NOR: Norfloxacin
S: Streptomycin
TE: Tetracycline
Spp: Species
