# Supplementary material for: The potential role of migratory birds in the transmission of pathogenic Campylobacter species to broiler chickens in broiler poultry farms and live bird markets
Source: BMC Microbiol. 2023 Mar 10;23:66. doi: 10.1186/s12866-023-02794-0 (PMC9999534; doi:10.1186/s12866-023-02794-0)
Supplement: Supplementary file 1 — Additional file 1: Table 1. oligonucleotide primers used for Campylobacter isolates characterization. Figure 1. Representative agarose gel electrophoresis of PCR products for Campylobacter isolates to detect 23S rRNA in genomic DNA. Lane L: DNA ladder, P: Positive control, N: Negative control and Lanes: 1 to 15 were positive samples. Figure 2. Representative agarose gel electrophoresis of PCR products for Campylobacter jejuni to detect mapA in genomic DNA. Lane L: DNA ladder, P: Positive control, N: Negative control and Lanes: 2, 8, 9, 10, 11, 12 ,13 and 14 were positive samples and Lanes: 1, 3, 4, 5, 6, 7 and 15 were negative. Figure 3. Representative agarose gel electrophoresis of PCR products for ceuE detection in genomic DNA. Lane L: DNA ladder, P: Positive control, N: Negative control and Lanes: 2, 8, 9, 10, 11, 12, 13 and 14 were negative samples and Lanes: 1, 3, 4, 5, 6, 7 and 15 were positive. Figure 4. Representative agarose gel electrophoresis of PCR products VirB11 detection in genomic DNA. Lane L: DNA ladder, P: Positive control, N: Negative control and Lanes: 1, 2, 5, 6, 7, 8, 9, 11, 12, 13, 14 and 15 were negative samples and Lanes: 3, 4, and 10 were positive. Figure 5. Representative agarose gel electrophoresis of PCR products for ciaB detection in genomic DNA. Lane L: DNA ladder, P: Positive control, N: Negative control and Lanes: 1, 2, 3, 4, 5, 8, 9, 10, 11 and 15 werenegative samples and Lanes: 6, 7, 12, 13 and 14 were positive. Figure 6. Representative agarose gel electrophoresis of PCR products for iam detection in genomic DNA. Lane L: DNA ladder, P: Positive control, N: Negative control and Lanes: 1 to 15 were positive. Figure 7. Representative agarose gel electrophoresis of PCR products to detect tetAin genomic DNA. Lane L: DNA ladder, P: Positive control, N: Negative control and Lanes: 1 to 15 were positive. Figure 8. Representative agarose gel electrophoresis of PCR products to detect BlaOXA-61 in genomic DNA. Lane L: DNA ladder, P: Positive [file 12866_2023_2794_MOESM1_ESM.docx]

**Supplementary table (1): oligonucleotide primers used for *Campylobacter* isolates characterization**

| **Target**  **gene** | **Primers sequences** | **Amplification**  **(bp)** | **Annealing temp.** | **Reference** |
| --- | --- | --- | --- | --- |
| *23S rRNA* | F: TATACCGGTAAGGAGTGCTGGAG | 650 | 55˚C  40 sec. | **[49]** |
|  | R: ATCAATTAACCTTCGAGCACCG |  |  |  |
| *C. coli*  *ceu*E | F: AAT TGA AAA TTG CTC CAA CTA TG | 462 | 58˚C  40 sec. | **[50]** |
|  | R: TGA TTT TAT TAT TTG TAG CAG CG |  |  |  |
| *C. jejuni*  *map*A | F: CTA TTT TAT TTT TGA GTG CTT GTG | 589 | 55˚C  40 sec. |  |
|  | R: GCT TTA TTT GCC ATT TGT TTT ATT A |  |  |  |
| *virB11* | F: TCTTGTGAGTTGCCTTACCCCTTTT | 494 | 53˚C  40 sec. | **[51]** |
|  | R: CCTGCGTGTCCTGTGTTATTTACCC |  |  |  |
| *iam* | F: GCGCAAATATTATCACCC | 518 | 55˚C  40 sec. | [**52]** |
|  | R: TTCACGACTACTACTATGCGG |  |  |  |
| *ciaB* | F: TGC GAG ATT TTT CGA GAA TG | 527 | 54˚C  40 sec. | [**53]** |
|  | R: TGC CCG CCT TAG AAC TTA CA |  |  |  |
| *tetA* | F: TTCTCTATATCGGGCGGATCGTGGC | 700 | 54˚C  40 sec. | **[54]** |
|  | R: CCACCCGAAGCAAGCAGGACCATG |  |  |  |
| *Bla*OXA-61 | F: AGAGTATAATACAAGCG | 372 | 54˚C  40 sec. | **55]]** |
|  | R: TAGTGAGTTGTCAAGCC |  |  |  |


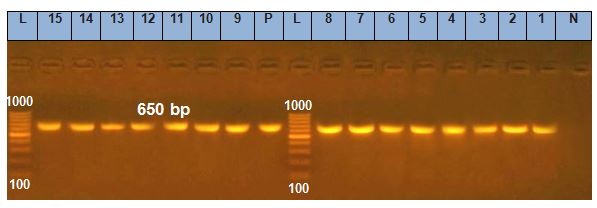


**Supplementary figure (1):** Representative agarose gel electrophoresis of PCR products for *Campylobacter* isolates to detect *23S rRNA* in genomic DNA. Lane L: DNA ladder, P: Positive control, N: Negative control and Lanes: 1 to 15 were positive samples.


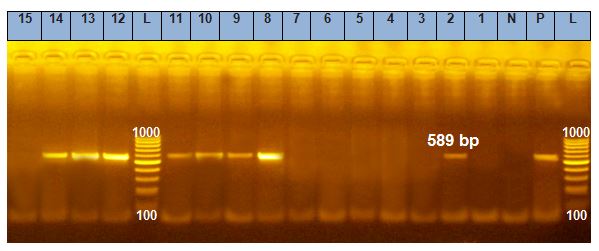


**Supplementary figure (2):** Representative agarose gel electrophoresis of PCR products for *Campylobacter jejuni* to detect *map*A in genomic DNA. Lane L: DNA ladder, P: Positive control, N: Negative control and Lanes: 2, 8, 9, 10, 11, 12 ,13 and 14 were positive samples and Lanes: 1, 3,4,5,6,7 and 15 were negative.


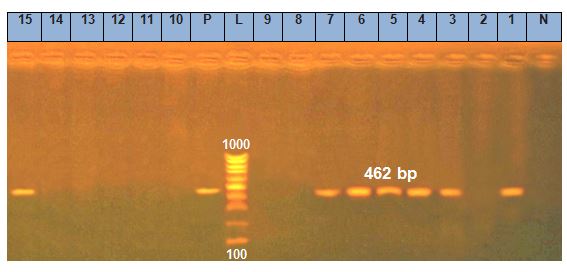


**Supplementary figure (3):** Representative agarose gel electrophoresis of PCR products for *ceu*E detection in genomic DNA. Lane L: DNA ladder, P: Positive control, N: Negative control and Lanes: 2, 8, 9, 10, 11, 12, 13 and 14 were negative samples and Lanes: 1, 3,4,5,6,7 and 15 were positive.


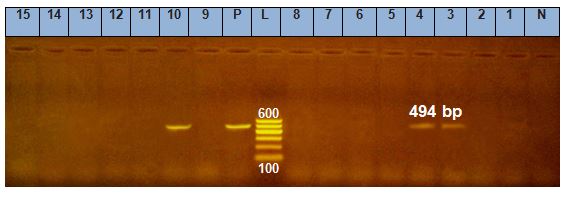


**Supplementary figure (4):** Representative agarose gel electrophoresis of PCR products *VirB11* detection in genomic DNA. Lane L: DNA ladder, P: Positive control, N: Negative control and Lanes: 1, 2, 5, 6, 7, 8, 9, 11, 12, 13, 14 and 15 were negative samples and Lanes: 3, 4, and 10 were positive.


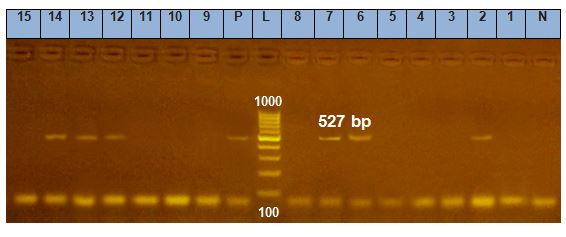
 **Supplementary figure (5):** Representative agarose gel electrophoresis of PCR products for *ciaB* detection in genomic DNA. Lane L: DNA ladder, P: Positive control, N: Negative control and Lanes: 1, 2, 3, 4, 5, 8, 9, 10, 11 and 15 were negative samples and Lanes: 6, 7, 12, 13 and 14 were positive.


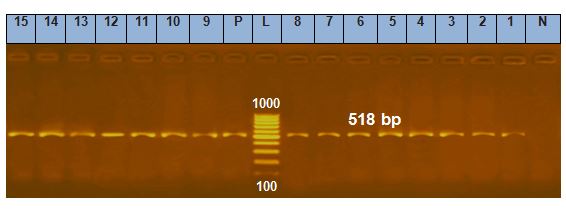
 **Supplementary figure (6):** Representative agarose gel electrophoresis of PCR products for *iam* detection in genomic DNA. Lane L: DNA ladder, P: Positive control, N: Negative control and Lanes: 1 to 15 were positive.


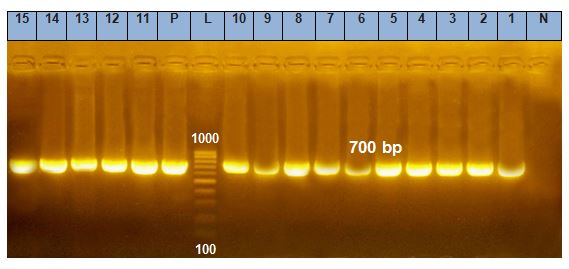


**Supplementary figure (7):** Representative agarose gel electrophoresis of PCR products to detect *tet*A in genomic DNA. Lane L: DNA ladder, P: Positive control, N: Negative control and Lanes: 1 to 15 were positive.


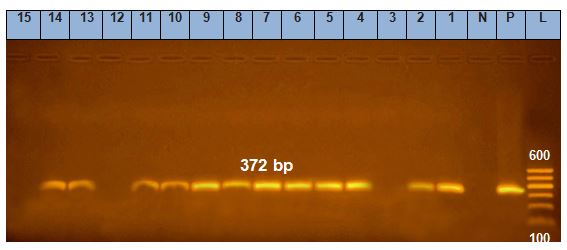


**Supplementary figure (8):** Representative agarose gel electrophoresis of PCR products to detect *BlaOXA-61* in genomic DNA. Lane L: DNA ladder, P: Positive control, N: Negative control and Lanes: 1, 2, 4, 5, 6, 7, 8, 9, 10, 11, 13 and 14 were positive and Lanes: 3, 12 and 15 were negative.


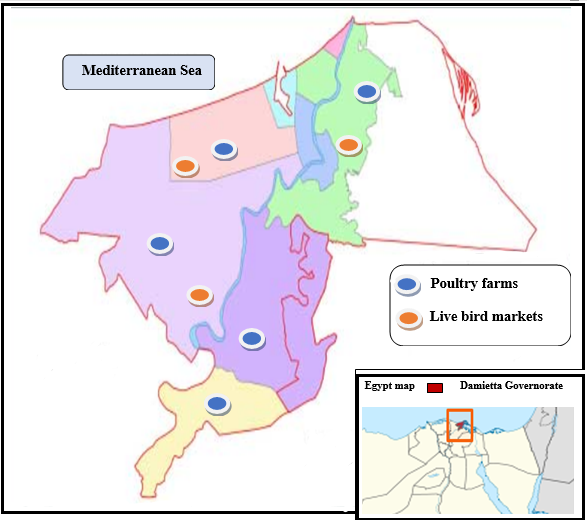


**Supplementary figure (9).** Map of Damietta Governorate showing the location of the selected five broiler chicken farms (blue circles represents poultry farms) and the three live bird markets (orange circles represents poultry farms) for the study in relation to the rest of Damietta Governorate.

**References**

**49. Wang G, Clark CG, Taylor TM, Pucknell C, Barton C, Price L, Woodward DL, Rodgers FG (2002).** Colony Multiplex PCR Assay for Identification and Differentiation of *Campylobacter jejuni*, *C. coli*, *C. lari*, *C. upsaliensis*, and *C. fetus* subsp*. Fetus*. JOURNAL OF CLINICAL MICROBIOLOGY, Vol. 40, No. 12. p. 4744–4747.

**50. Eunju S, Lee Y (2009).** Comparison of Three Different Methods for *Campylobacter* Isolation from Porcine Intestines. J. Microbiol. Biotechnol., 19(7), 647–650.

**51**. **Datta S, Niwa H, Itoh K. (2003).** Prevalence of 11 pathogenic genes of *Campylobacter jejuni* by PCR in strains isolated fromhumans, poultry meat and broiler and bovine faeces.Journal of Medical Microbiology (2003), 52, 345–348 DOI 10.1099.

**52**. **Wieczorek, K.; Szewczyk, R. and Osek, J. (2012):** Prevalence, antimicrobial resistance, and molecular characterization of *Campylobacter jejuni* and *C. coli* isolated from retail raw meat in Poland. *Veterinarni Medicina, 57, 2012 (6): 293–299.*

**53.** [**Zheng J**](https://www.ncbi.nlm.nih.gov/pubmed/?term=Zheng%20J%5BAuthor%5D&cauthor=true&cauthor_uid=16629018)**,**[**Meng J**](https://www.ncbi.nlm.nih.gov/pubmed/?term=Meng%20J%5BAuthor%5D&cauthor=true&cauthor_uid=16629018)**,**[**Zhao S**](https://www.ncbi.nlm.nih.gov/pubmed/?term=Zhao%20S%5BAuthor%5D&cauthor=true&cauthor_uid=16629018)**,**[**Singh R**](https://www.ncbi.nlm.nih.gov/pubmed/?term=Singh%20R%5BAuthor%5D&cauthor=true&cauthor_uid=16629018)**,**[**Song W**](https://www.ncbi.nlm.nih.gov/pubmed/?term=Song%20W%5BAuthor%5D&cauthor=true&cauthor_uid=16629018)**. (2006):** Adherence to and invasion of human intestinal epithelial cells by *Campylobacter jejuni* and *Campylobacter coli* isolates from retail meat products. [J Food Prot.](https://www.ncbi.nlm.nih.gov/pubmed/16629018);69(4):768-74.

**54.** [**Gibreel A**](https://www.ncbi.nlm.nih.gov/pubmed/?term=Gibreel%20A%5BAuthor%5D&cauthor=true&cauthor_uid=15328109)**, [Tracz DM](https://www.ncbi.nlm.nih.gov/pubmed/?term=Tracz%20DM%5BAuthor%5D&cauthor=true&cauthor_uid=15328109),**[**Nonaka L**](https://www.ncbi.nlm.nih.gov/pubmed/?term=Nonaka%20L%5BAuthor%5D&cauthor=true&cauthor_uid=15328109)**,**[**Ngo TM**](https://www.ncbi.nlm.nih.gov/pubmed/?term=Ngo%20TM%5BAuthor%5D&cauthor=true&cauthor_uid=15328109)**,**[**Connell SR**](https://www.ncbi.nlm.nih.gov/pubmed/?term=Connell%20SR%5BAuthor%5D&cauthor=true&cauthor_uid=15328109)**,** [**Taylor DE**](https://www.ncbi.nlm.nih.gov/pubmed/?term=Taylor%20DE%5BAuthor%5D&cauthor=true&cauthor_uid=15328109) **(2004).** Incidence of antibiotic resistance in *Campylobacter jejuni* isolated in Alberta, Canada, from 1999 to 2002, with special reference to tet (O)-mediated tetracycline resistance. Antimicrob Agents Chemother., Sep;48(9):3442-3450.

**55. Sierra-Arguello YM, Morgan RB, Perdoncini G, Lima LM, Gomes MJP, do Nascimento VP (2015).** Resistance to β-lactam and tetracycline in *Campylobacter* spp. isolated from broiler slaughterhouses in southern Brazil. Pesq. Vet. Bras. 35(7):637-642.
